# Preclinical characterization of therapeutic antibodies targeted at the carboxy-terminus of Sonic hedgehog

**DOI:** 10.18632/oncotarget.24510

**Published:** 2018-02-16

**Authors:** Bhairavi Tolani, Ngoc T. Hoang, Luis A. Acevedo, Etienne Giroux Leprieur, Hui Li, Biao He, David M. Jablons

**Affiliations:** ^1^ Thoracic Oncology Program, Department of Surgery, Helen Diller Family Comprehensive Cancer Center, University of California, San Francisco, CA, USA; ^2^ Respiratory Diseases and Thoracic Oncology Department, APHP-Ambroise Pare Hospital, Boulogne-Billancourt, France

**Keywords:** sonic hedgehog (Shh), cancer stem cells (CSCs), therapeutic antibody, targeted therapy, non-small cell lung cancer (NSCLC)

## Abstract

The Sonic Hedgehog (Shh) signaling pathway has been implicated in the development and tumor progression of a number of human cancers. Using synthetic peptide mimics to mount an immune response, we generated a mouse mAb to the carboxy (C)-terminus of the Shh protein and characterized its preclinical antitumor effects. *In vitro* screening guided selection of the best candidate for mAb scale-up production and therapeutic development. C-term anti-Shh, Ab 1C11-2G4 was selected based on ELISA screens, Western blotting, and flow cytometric analyses. Purified Ab 1C11-2G4 was shown to recognize and bind both Shh peptide mimics and cell surface Shh. Administration of Ab 1C11-2G4 not only reduced cell viability in 7 cancer cell lines but also significantly inhibitted tumor growth in a xenograft model of A549 lung cancer cells. *Ex vivo* analyses of xenograft tumors revealed a reduction in Shh signal transduction and apoptosis in 2G4-treated mice. Collectively, our results provide early demonstration of the antitumor utility of antibodies specific for the C-terminal region of Shh, and support continued development to evaluate their potential efficacy in cancers in which Shh activity is elevated.

## INTRODUCTION

Dysregulations in the Sonic Hedgehog (Shh) signaling pathway, such as constitutive overexpression of the Shh ligand, have been implicated in tumorigenesis in a number of tumor types [1–6]. When ectopically expressed, Shh triggers tumor formation in preclinical models [7–8], lending credence to the idea that ligand-dependent Shh pathway activation promotes tumor initiation and progression. Targeting ligand-dependent Shh signaling with Smoothened antagonists [9–12] or Shh neutralizing antibodies [13–15] inhibits the growth of several tumor cell lines *in vitro* and *in vivo* [16–18]. One Smoothened antagonist, Vismodegib, was granted FDA approval in 2013 for the treatment of metastatic basal cell carcinoma, an Shh ligand-independent malignancy. However, clinical progress against other Shh ligand-dependent cancers has been disappointing [19]. Currently, other small molecule inhibitors of Smoothened, such as Sonidegib, BMS-833923, Glasdegib, and Taladegib, are under clinical investigation for co-administration with chemotherapeutic agents for treatment in various cancers. However, acquired resistance to pathway-targeted drugs due to compensatory mutations is frequent and can lead to tumor relapse. Cyclopamine-related molecules, which also target Smoothened, have also been limited by non-specific off-target effects at high concentrations.

In contrast, therapeutic antibodies are stringent binders to their intended targets. An amino (N)-terminal Shh mouse monoclonal antibody, 5E1, has been used as a research tool to study Hedgehog biology by specifically blocking ligand-dependent pathway activation [14]. In 2014, the first fully human Hedgehog antibody, MEDI-5304, specific for full-length Hedgehog was reported, but had no effect as a single agent or when combined with chemotherapy on cancer stem cell (CSC) frequency, thought to be regulated by Hedgehog activity, or on *in vitro* growth of primary pancreatic cancer explants [15]. The combination of an anti-Shh antibody with anti-CD47 antibodies in the treatment of bladder cancer has been reported [20]. The small molecule inhibitors GANT58/GANT61 [21] and Robotnikinin target Hedgehog pathway proteins GLI and Shh, respectively, but have exhibited only limited *in vitro* suppression of Shh transcriptional activity or cell growth [22, 23].

The full-length Shh protein is cleaved in the cytoplasm into N- and C-terminal fragments. The N-terminus of Shh is required as a ligand for biological development, but no known function of the secreted C-terminus has been reported. Upon ligand binding, the N-terminal Shh protein fragment relieves the inhibition of transmembrane protein Patched on Smoothened to activate the Shh signaling pathway via transcriptional activation of GLI [24–26]. This pathway is crucial for embryonic development and when aberrantly reactivated, has been implicated in multiple cancers including non-small cell lung cancer (NSCLC) [27–30]. While blocking the Shh pathway is an attractive anti-cancer strategy, no therapeutic antibody raised against the carboxy (C)-terminal of the Shh protein has been described. This could be because the cleaved C-terminus has no known signaling function and thus no efforts have been made to target it therapeutically. We recently reported the presence of uncleaved, membrane-bound, full-length Shh (Shh+) on fresh primary lung tumors collected from patients [31] and in cancer cell lines, and have reported initial validation of this full-length protein as a novel marker of cancer stem cells (CSCs) in NSCLC. This Shh+ subpopulation (~1%) of cells displays characteristics of CSCs, which are thought to drive tumor initiation, maintenance and even survival post-chemotherapy via ligand-dependent paracrine mechanisms [32–33]. We also showed that pharmacological inhibition of the Shh pathway in these Shh+ cells suppresses their CSC features. These findings led us to hypothesize that antibodies generated against the C-terminus Shh epitope can bind and neutralize full-length Shh found exclusively on the CSC population, while leaving the cleaved N-terminus Shh, important for physiologic Shh signaling, unperturbed. Thus, we sought to use C-terminus anti-Shh antibodies as a novel treatment stratagy to specifically target full-lenghth Shh present on CSCs.

## RESULTS

### Generation of a repertoire of novel therapeutic antibody candidates targeted at C-terminal Sonic hedgehog (Shh) derived from mouse hybridoma clones

**Figure 1:**
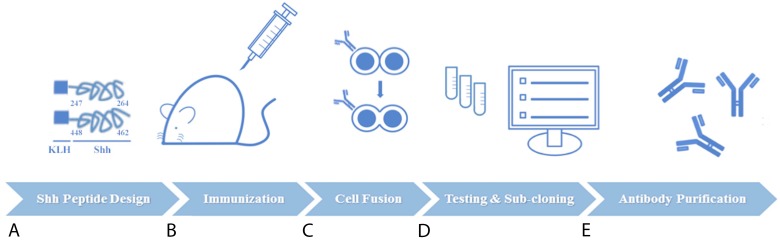
Schematic of experimental procedures for generation of novel anti-Sonic Hedgehog (Shh) candidate therapeutic antibodies directed at the carboxy (C)-terminal (**A**) Two synthetic peptide mimics of Shh (AA 247-264 and AA 448-462) were conjugated to carrier protein KLH and used as the immunogen. (**B**) Dual administration of both peptides was used to elicit an immune response in Sp2/0-Ag1 mice. (**C**) B cells from immunized murine lymph nodes were isolated and fused to myeloma cells for hybridoma generation. (**D**) Protein-based screening analyses guided the selection of the best hybridoma clones followed by one round of sub-cloning to isolate monoclonal antibody-producing hybridoma cells (**E**) Large-scale preparations of purified antibodies from hybdridomas were used for *in vitro*, *in vivo*, and *ex vivo* investigations.

We constructed a novel murine repertoire of antibody candidates derived from hybridoma clone fusions isolated from the lymph nodes of Sonic hedgehog-immunized mice. Specialized antigen-designing software was used to construct two synthetic peptide mimics of the C-terminal human Sonic hedgehog protein: 1) Shh 247-264 AA and 2) Shh 448-462 AA (Figure 1A). Following dual intravenous (IV) administration of both KLH-conjugated peptides into Sp2/0-Ag14 mice to mount an immune response (Figure 1B), the B-cells from the isolated lymph nodes were fused to myeloma cells to generate a diverse repertoire of hybridomas (Figure 1C).We next performed protein-based screening analyses for anti-Shh C-term therapeutic antibody hybridoma candidates by capture ELISA, Western blotting and flow cytometry using both cancer cells and cells exogenously transfected for the expression of Shh (Figure 1D). One hybridoma clone from this screening strategy, 1C11, was isolated, sub-cloned and subjected again to the same selection procedure as the parental clones using ELISAs, Western blots and flow cytometric evaluations. Large-scale protein purification of antibodies from 1C11 hybridoma sub-clones designated 2G4 and 2D9 yielded sufficient protein required for *in vitro* and *in vivo* analyses (Figure 1E).

### Screening and selection of precursor antibodies directed at C-terminal Shh

**Figure 2:**
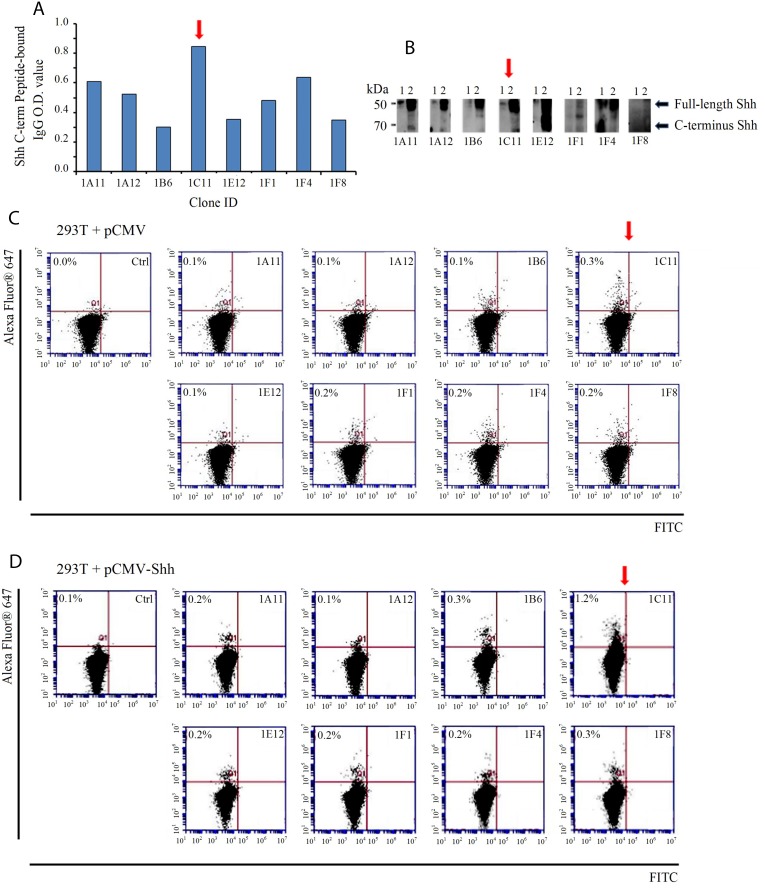
Screening of murine hybridoma clones for selection of candidate therapeutic anti-Shh antibodies directed at the C-terminal of the Sonic Hedgehog protein (**A**) Supernatants from antibody- producing hybridoma clone cells were incubated with synthetic peptide mimics of Shh as measured by an ELISA-based assay. Clone 1C11 (red arrow) showed the strongest binding to the same Shh peptide mimics used to generate an immune response. (**B**) Hybridoma clonal supernatants were incubated with the following cell lysates to check for Shh protein detection and analyzed by Western blotting: 1) 293T (endogenous Shh) and 2) 293T (exogenously transfected with pCMV-Shh) (**C**) Flow cytometric evaluation of cell-surface Shh recognition performed with clonal supernatants incubated with non-permeabilized 293T cells containing the vector control or (**D**) pCMV-Shh. Clone 1C11 detected the highest percentage of cell-surface Shh compared with the other clones. The secondary antibody alone incubated with cells was run as a negative control.

To select the best C-term Shh antibodies from over 50 hybridoma clones we designed two sets of screening assays. First, we employed the same Shh C-term peptides used to mount an immune response in mice (AA 247-264 and 448-462, KLH-free version) in an ELISA-based assay with supernatants from antibody-producing hybridoma clonal cells; hybridoma 1C11showed the highest binding (Figure 2A). Next, we utilized two cell lines, 293T expressing endogenous Shh and 293T transfected with exogenously Shh, in both Western blotting and flow cytometry. Consistent with our ELISA results, clone 1C11 proved to be the most effective at detecting Shh bands in cell lysates via Western blotting (Figure 2B). Flow cytometry demonstrated that 1C11 recognizes and binds endogenous cell-surface Shh in 0.3% of 293T cells bearing the vector control (Figure 2C) and exogenous Shh in 1.2% of CMV-Shh transfected 293T cells (Figure 2D) more strongly than other clones.

### *In vitro* characterization of IC11 sub-clones

**Figure 3:**
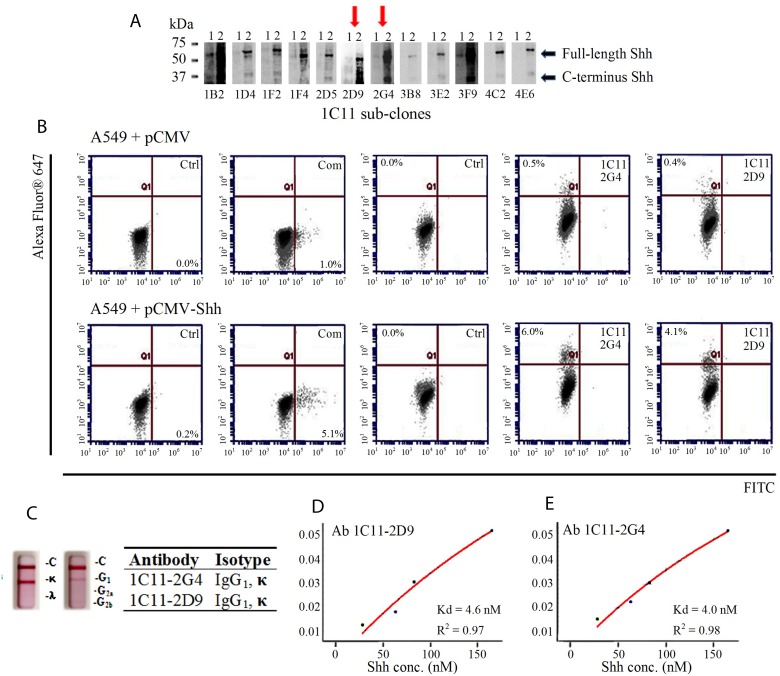
Characterization of 1C11 sub-clones (**A**) Supernatants of 12 sub-clones derived from parental clone 1C11 were incubated with 2 different cell lysates to check for Shh epitope recognition by Western blotting: 1) 293T (endogenous Shh-vector control) and 2) 293T (exogenously transfected with pCMV-Shh) (**B**) Flow cytometric evaluation of Shh recognition performed with purified antibodies Ab 1C11-2G4, Ab 1C11-2D9 and a commercial (Com) C-term antibody on vector control (pCMV) A549 cells (top panel) and A549 cells transfected with pCMV-Shh (bottom panel) compared with their respective negative controls of cells incubated with secondary antibody alone (Ctrl.). (**C**) Two sub-clones of 1C11, 2G4 and 2D9, exhibit IgG_1_, kappa measured using an isotyping kit. Binding affinities (K_d_) of antibodies Ab 1C11-2D9 (**D**) and Ab 1C11-2G4 (**E**) for recombinant Shh protein.

Parental Ab 1C11-producing hybridoma cells were sub-cloned in an iterative effort to isolate monoclonal antibodies with stronger binding properties, and the 12 resulting sub-clones were tested in the same type of screening assays described above. The supernatants from sub-clones 2D9 and 2G4 showed Shh recogniztion by Western blotting (Figure 3A) and flow cytometry with 293T exogenously transfected with Shh (Supplementary Figure 1); 0.91% in 2D9 and 0.95% in 2G4. Purified monoclonal antibodies 1C11-2G4 and 1C11-2D9 were used for the subsequent biolayer inferometry (BLI) binding studies, isotyping and flow cytometric evaluation in A549 cells to confirm specificity using the same C-term Shh peptides for which they were raised. To confirm specific recognition, A549 (+vector control) cells and A549 cells transfected with exogenous Shh were labeled with 2G4 and 2D9 and assessed via flow cytometry. Both sub-clones recognized endogenous Shh (Figure 3B, top panel) and exogenous Shh transfected in A549 cells (Figure 3B, bottom panel) at a level similar to that of a commercially available C-terminus anti-Shh antibody. Also, Abs 1C11-2G4 and 1C11-2D9, were both isotyped as IgG_1_, κ (Figure 3C). To further characterize the binding properties in a cell-free system using Shh peptides, we covalently bound and immobilized both candidate and control antibodies on amine-reactive sensor tips using an Octet RED 384 system). Upon introduction of increasing concentrations of Shh peptide-mimic ligands (Shh 247-264 AA and Shh 448-462 AA), we observed that both Abs 1C11-2G4 and 1C11-2D9 resulted in increased binding that was not observed with the IgG control antibody (data not shown). We calculated the K_d_ values of both antibodies with recombinant Shh and observed nanomolar binding affinities (Figure 3D and 3E). Taken together, these results confirm the specificity of our in-house Shh C-term antibodies for the Sonic Hedgehog protein.

### Ab 1C11 C-term Shh antibodies recognize Shh+ expressing cell populations and inhibit the growth of cancer cells *in vitro*

**Figure 4:**
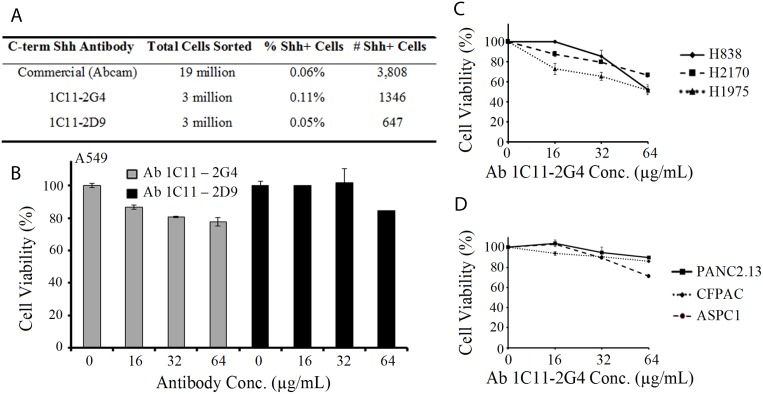
Anti-Shh antibodies directed at the C-terminal of the Sonic Hedgehog protein recognize Shh+ cell populations and reduce cell viability of cancer cells in a dose-dependent manner (**A**) Fluorescence Activated Cell Sorting (FACS) of A549 cells labeled with Ab 1C11-2G4, Ab 1C11-2D9 and commercially purchased (Abcam) anti-Shh antibodies showing the % and number of Shh+ cells sorted. (**B**) NSCLC A549 cells were treated with indicated doses of C-term anti-Shh 1C11-2G4 and 1C11-2D9 antibodies (reconstituted in PBS) in triplicate for 96 hours and cell viability/ATP content was measured using CellTiter-Glo Luminescent Cell Viability Assay. PBS was used as a negative control. The results shown for all cell viability figures are representative of two independent experiments. Error bars indicate means ± S.D. Effect of Ab 1C11-2G4 on cell viability in lung cancer (**C**) and pancreatic cancer (**D**) performed as in (B).

To corroborate that our two anti-Shh antibodies 1C11-2G4 and 1C11-2D9 recognize cell-surface expression of Shh, we performed FACS analysis on labeled A549 cells without membrane permeabilization. Sub-clone Ab 1C11-2G4 recognized a higher number of Shh+ cells from a mixed population (0.11%) than either 1C11-2D9 (0.05%) or a commercially available C-terminus antibody (0.06%, Abcam) (Figure 4A). Our studies show that *in vitro* delivery of purified 1C11 anti-C-terminus Shh antibodies decreased NSCLC A549 tumor cell proliferation as evidenced by a modest reduction of cell viability compared to treatment with control antibody, and that sub-clone 2G4 showed a marginally stronger anti-proliferative capacity than 2D9 (Figure 4B). Furthermore, we observed that Ab 1C11-2G4 also inhibited the growth of other lung and pancreatic cancer cell lines (Figure 4C and Figure 4D) reported to possess CSC populations [34–38]. Given the small percentage of Shh+ cells present in these cell lines, we observed a modest antiproliferative effect of our therapeutic antibodies. However, when C-terminal directed anti-Shh antibodies were combined with targeted therapy (our in-house Gli inhibitor, Gli-I or Vismodegib, Supplementary Figure 2A–2C) or chemotherapy, (Docetaxel Supplementary Figure 2D), these combinatorial regimens showed increased efficacy. Based on these collective observations, Ab 1C11-2G4 was advanced for further analyses in *in vivo* animal studies.

### Reduction of tumor burden upon *in vivo* xenograft treatment with C-terminus Shh antibodies in mice

**Figure 5:**
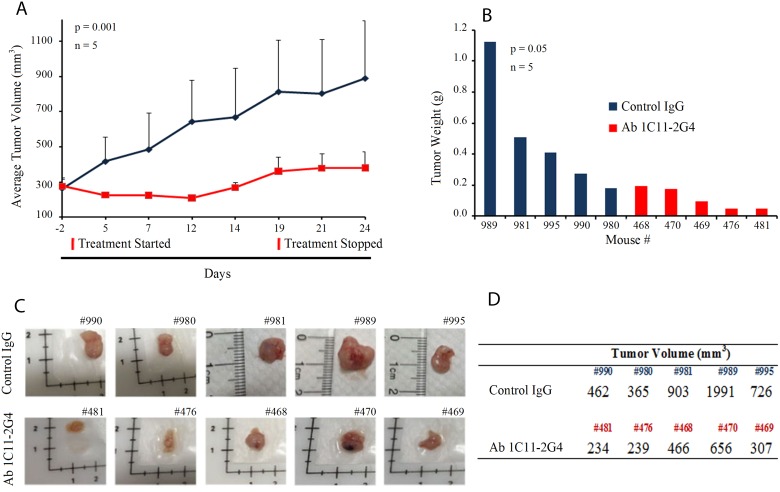
C-term Shh Ab 1C11 – 2G4 treatment inhibits *in vivo* growth of NSCLC in a mouse xenograft model (**A**) 7-week-old female nude mice were inoculated with 10 × 10^6^ NSCLC A549 cells in the flank region to establish tumors and randomly assigned to treatment regimens of IgG control or Ab 1C11-2G4 (8 mg/kg × 3 times a week for 3 weeks, intratumorally). Changes in tumor volume of mice treated with the antibodies IgG control or Ab 1C11 – 2G4 (*n* = 5, *p* = 0.001) over the course of the study are shown. At the end point, tumor mass (**B**) was determined on harvested tumors by weighing (*n* = 5, *p* = 0.05). (**C**) Images of tumors collected from antibody-treated mice with their identifying ear-tag mouse numbers (#) showing a clear reduction in tumor volumes (**D**) calculated using the formula (V = L × W^2^) in Ab 1C11 – 2G4-treated mice when compared with the control IgG antibody.

To determine the *in vivo* therapeutic utility of our C-terminus Shh antibody, we injected either Ab 1C11-2G4 or control IgG antibodies into the tumors of mice bearing A549-derived NSCLC xenografts. Mice were randomly assigned to control antibody (*n* = 5) or therapeutic antibody-treated groups (*n* = 5, 8 mg/kg intratumorally, 3 times a week for 3 weeks). During the course of the study, all mice survived but tumor volume decreased significantly in the Ab 1C11-2G4-treated mice (Figure 5A, *p* = 0.001). Comparative analysis of harvested tumors clearly exhibited a decrease in tumor weight (Figure 5B, *p* = 0.05), size (Figure 5C), and tumor volume (Figure 5D) in the C-terminus Shh antibody-treated group. Similar body weights in both treatment groups suggested a lack of toxicity from therapeutic antibody treatment (Supplementary Figure 3A). Collectively, these results indicate that anti-C-terminus Shh antibodies may be both safe and efficacious in reducing tumor volume (Supplementary Figure 3A and 3B).

### Ab1C11-2G4 treatment down-regulates Gli and induces apoptosis

**Figure 6:**
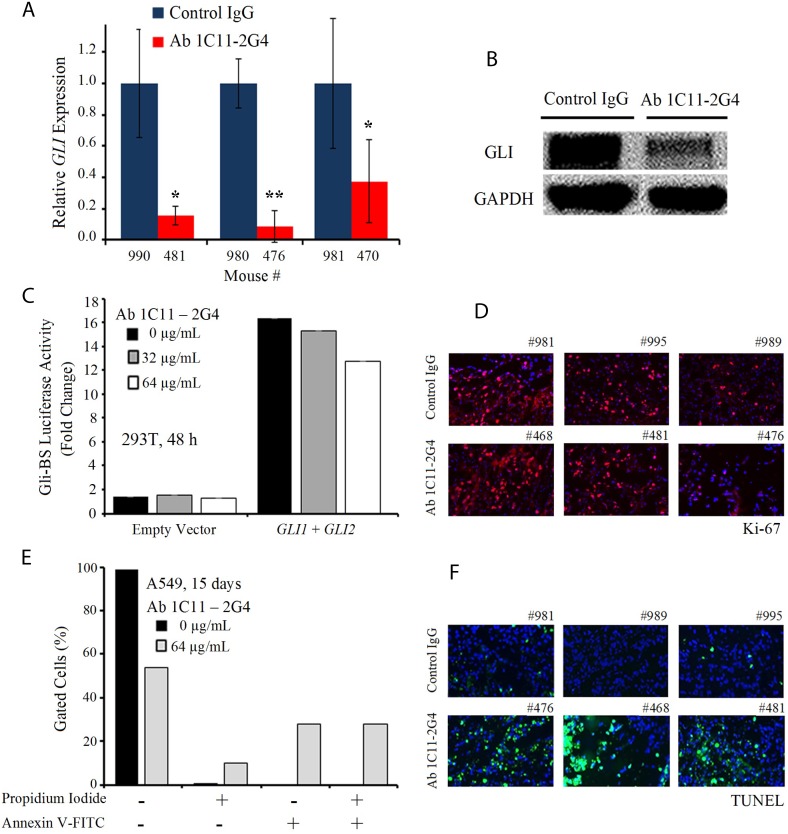
C-term Shh Ab 1C11–2G4 treatment down-regulates Gli and induces apoptosis (**A**) *Ex vivo* quantitative RT-PCR analysis of *GLI* levels in A549 harvested tumors treated with IgG controls OR 8 mg/kg Ab 1C11-2G4, 3× per week for 3 weeks. Real-time PCR reactions were performed in triplicate and the data presented as fold change in target gene expression (mean ± SD) after normalization with GAPDH. The results shown are representative of two independent experiments. (**B**) Corresponding tumors after control IgG OR Ab 1C11-2G4 treatments were lysed and analyzed by Western blot for the expression of GLI. GAPDH was used as a loading control. (**C**) Empty vector OR *GLI1* and *GLI2*-induced transcriptional activation in 293T cells left untreated or treated with 2 doses of Ab 1C11-2G4 for 48 hours. An expression construct linking the 8 repeats of Gli-binding sites (Gli BS) to a luciferase reporter was used as a surrogate measurement of the Gli-dependent transcription. All measured luciferase activities were normalized to pRL-TK vector activity. The data represent means ± S.D. (**D**) Control IgG- or Ab 1C11-2G4-treated tumor sections stained for a common proliferation marker, Ki-67. Representative images captured at 20× are presented. (**E**) A549 cells were treated with 64 μg/mL of Ab 1C11-2G4 for 15 days, stained with Annexin V-FITC/PI and analyzed for apoptosis by flow cytometry. The resulting percentages are presented as a graph. (**F**) Control IgG- or Ab 1C11-2G4-treated tumor sections stained for apoptosis induction via a TUNEL assay. Representative images captured at 20× are presented.

C-terminal anti-Shh antibody 1C11-2G4 inhibited tumor growth in mouse xenografts of lung cancer *in vivo*, suggesting that our novel therpeutic may successfully target Shh and cell-mediated hedgehog signalling via C-term epitope binding. We next sought to confirm whether functional targeting of the Shh protein impaired Shh signal transduction programs in Ab 1C11-2G4-treated tumors. To test the effect of antibody treatment on signaling of Shh-mediated transcription factor GLI mRNA and protein expression levels, we performed qRT-PCR and Western blot analyses, respectively, with tumor lysates and observed a significant reduction in *GLI* transcripts (Figure 6A) and GLI polypeptide expression (Figure 6B) in samples, *ex vivo*, compared with the IgG controls. Next, to confirmed the functional significance of Ab 1C11-2G4 treatment, we investigated the effect of co-transfection of *GLI1* and *GLI2* on Gli-dependent transcription activity in 293T cells after 48 hours, which is a relatively short period of time given our hypothesis that the antibody targets Shh+ cells. We observed a mild dose-dependent decrease in the levels of transcription of a Gli-driven luciferase construct (Gli-Luc) compared with the empty vector control in Ab 1C11-2G4-treated cells (Figure 6C). We next stained the antibody-treated tumor sections with a commonly used proliferation marker, Ki-67, and observed only a mild difference relative to the IgG control (Figure 6D), which was consistent with findings shown in Figures 4B, C and D. To determine the mechanism by which our therapeutic antibodies caused cell death, we stained A549 cells with Annexin V and propidium iodide (PI) after 15 days of antibody treatment. We observed an increase in the proportion of apoptotic cells upon Ab 1C11-2G4 treatment, indicating induction of cell death via apoptosis (Figure 6E). Consistent with it, treatment with Ab 1C11-2G4 resulted in a modest increase in Annexin V^+^/PI^+^ cells. Finally, to confirm cell death via apoptosis in control IgG and Ab 1C11-2G4-treated tumors we used a TUNEL assay. The much higher TUNEL-positive staining we observed in antibody-treated tissues was indicative of enhanced apoptosis induction (Figure 6F). Taken together, these results support the hypothesis that modest down-regulation of the Shh pathway occurs but apoptosis is the key mechanism of the anti-tumor activity observed with binding of Shh protein by Ab 1C11-2G4.

## DISCUSSION

Targeted therapeutic antibodies have become important anti-cancer agents. Overexpression of the Shh ligand has been implicated in the development of several cancers [1–6], and therapeutics aimed at eliminating pathological Shh signaling may benefit the treatment of a number of tumor types that are Shh-mediated via ligand-dependent autocrine and paracrine mechanisms [9–18].

Even though the Shh pathway is mainly quiescent in adults, the safety of Shh-targeting with therapeutic antibodies was questioned initially because not only does the N-term of the Shh protein play an important role in embryonic development, but Shh also plays a poorly understood role in tissue homeostasis and repair in adults [27–30]. However, no signaling function of the cleaved C-terminus of the protein has been reported, suggesting that targeting the C-terminus of secreted Shh or even rare cases of the un-cleaved, full-length Shh protein found on CSCs may be a safer strategy for antibody-mediated reduction of Shh-pathology [39–41]. We recently reported the presence of membrane-bound full-length Shh protein in human NSCLC cells and showed that these Shh positive (Shh+) cells (~1% of the total tumor cell population) harbored CSC-like characteristics [31]. Furthermore, Shh+ cells expressing the full-length protein appeared to provid a signal for proliferation, migration and chemotherpy-resistance properties, making functional targeting of full-length Shh by the use of C-term anti-Shh antibodies an attractive therapeutic strategy.

In this study, we report the development and characterization of a novel C-terminal anti-Shh antibody, Ab 1C11-2G4, selected and purified from a screen of over 50 antibody-producing hybridoma candidates derived from murine clones. We screened mouse hybridomas based on their ability to bind to recombinant fragments of Shh and Shh-expressing cells in both endogenous and exogenous systems. We demonstrated proof-of-principle that the therapeutic anti-Shh antibody Ab 1C11-2G4 recognizes and binds its target, Shh protein. In particular, Ab 2G4 recognizes and binds not only the Shh peptide via biolayer inferometry and possesses nanomolar affinity for its ligand, but also recognizes cell-surface Shh+ expressing cell populations and inhibits the viability of several cancer cell lines *in vitro.* We attribute the modest anti-proliferative effect of 2G4 to the fact that CSC populations tend to be very low (~1%) and we previousy reported the real % Shh+ cell population as ~0.15% [31] , and thus for the time scale used here (96-hours), we expect that inhibiting Shh signaling in CSCs will not result in dramatic changes in viability. In addition, in our studies using Ab 1C11-2G4 in an Shh-expressing xenograft model of lung cancer, local delivery of 2G4 over a few weeks had an anti-proliferative effect on A549 tumors. *Ex vivo* analyses of A549 xenograft tumors from mice treated with the C-term Shh antibody Ab 1C11-2G4 provided further support that Shh signal transduction is modestly down-regulated after treatment, as evidenced by suppressed transcript and protein levels of Shh downstream target GLI. However, cell death via apoptosis appears to be the primary mechanism of the therapeutic antibody.

The results of our study highlight a new therapeutic avenue of blocking the protein-protein interaction interface for regulating Shh signaling by use of anti-Shh antibodies directed at the C-term as a way to target full-length Shh, which our recent studies have implicated as the ‘signal source’ of CSCs. Activation of the pathway occurs when Shh binds to its receptor Patched, but signaling is inhibited in a small population of cells if full-length Shh binds instead to the blocking antibody 2G4, a potential therapeutic, so that the protein cannot transmit downstream signals. We believe that the modest efficacy of our C-term anti-Shh antibody is associated with the fact that only a very small population (~1%) of tumor cells express full-length Shh [31]. Thus, sustained treatment with 2G4 over time could be used to eradicate CSC-like Shh+ cells in Shh-dependent tumor types without severe toxicity. Another reason for the modest efficacy we observed could be that our antibody also binds cleaved C-term Shh produced by a majority of the cells (~99%), which has no known Shh signaling function, and which may deplete the amount of antibody available for therapeutic binding to cell-surface bound full-length Shh.

To our knowledge, our study of Ab 1C11-2G4 is the first report of a therapeutic and inhibitory antibody designed to specifically target the carboxy-terminus of Shh. Although several commercially available C-term antibodies are available, and we used one as a positive control in our studies, none have been develooped as a therapeutic modality. Other anti-Shh antibodies are under investigation as anti-tumor agents [15, 20], including one known to target full-length Shh; none have been reported to alter CSC populations. Our studies have established that 2G4 shows modest anti-tumor activity *in vitro* and *in vivo* with a lack of obvious toxicity; it may act by targeting a population of full-length-Shh+ expressing CSC cells [31]. This novel strategy may prove efficacious in further pre-clinical translational studies.

## MATERIALS AND METHODS

### Generation and purification of monoclonal IgG antibodies

A proprietary algorithm (ThermoFisher) was used to analyze amino acid sequences and suggest the best small peptide sequences for antibody generation and development. The algorithm took into account specific structural motifs, charge, hydrophylicity and historically successful sequences. Two synthetic peptide mimics of the C-terminal human Sonic Hedgehog Protein (C-term: 198-462 AA) were selected: 1) Shh 247-264 AA and 2) Shh 448-462 AA. Both peptides were KLH-conjugated and injected as intraperitoneal emulsions in Freund's Complete Adjuvant (CFA) into Sp2/0-Ag14 mice to mount an immune response (primary and first-booster). An ELISA employing the KLH free peptides as an antigen was used to confirm the production of the antibodies in the sera of the mice. Two mice with the strongest response were advanced into the fusion phase with myeloma cells in which the lymphocytes from the spleen/lymph nodes were seeded in 96-well plates with rich growth medium. Cell culture supernatants from proliferating colonies were screened against the free peptide antigens and full-length Shh both in 1) endogenous 293T cells and 2) exogenously transfected 293T + pCMV-Shh cells via flow cytometry and Western blotting. Expression of full-length Shh polypeptide in transfected cells increased the percentage of cells labeled with the Shh antibodies produced by two sub-clones (Ab 1C11-2D9 and Ab 1C11-2G4) identified after the screening, compared to the percentage of un-transfected cells labeled by the same antibodies, as measured by flow cytometry. Positively identified parental cell clones were re-plated to segregate into sub-clones followed by the above-mentioned screening procedure. About 51 clones and sub-clone producing antibodies were raised against the C-terminal Shh peptides. Finally, two selected sub-clones, 1C11-2G4 and 1C11-2D9, were advanced to large-scale purification via chromatography of monoclonal antibodies from hybridoma cells, which recognize the C-terminal region of human Shh. These were used for subsequent *in vitro* and *in vivo* experimentation. The antibodies raised against the C-terminal region of the Shh peptides are covered by patent number 62/356,276 filed with the USPTO.

### Octet binding studies

Purified Ab 1C11-2D9, Ab 1C11-2G4, or control IgG antibodies in 1× HBS were covalently attached to amine-reactive second-generation (AR2G) biosensor tips (ForteBio) along with an amine coupling kit (GE Healthcare) by following the manufacturer's recommendations on an Octet RED 384 machine (ForteBio, PALL Octet System). Antibody immobilization was checked via OctetÒ Software prior to introduction of increasing concentrations (37, 111, 333, 1000, 3000 nM) of Shh peptide-mimic ligands (Shh 247-264 AA and Shh 448-462 AA). Recombinant Shh proteins were purchased from commercial vendors (Abcam, e-Bioscience). Graphical output from the Octet^®^ Software of representative data from two independent experiments is presented.

### ELISA screen

KLH-free Shh peptides used for antibody generation were used as the antigen and coated onto plastic ELISA plates followed by incubation with sera containing antibodies from immunized mice or cell culture supernatants. After PBS washes and the addition of secondary antibodies, the optical density (O.D.) values produced as a result of binding/TMB substrate system for ELISA were recorded.

### Western blot analysis

Total protein from whole-cell lysates was prepared with M-PER buffer (ThermoFisher Scientific) and from frozen tissues with T-PER (ThermoFisher Scientific) supplemented with protease and phophatase inhibitors (Roche). Subsequently protein concentrations were determined via a Bradford assay. Equal quantities of proteins were combined with 5X protein loading buffer and separated by SDS-PAGE followed by PVDF membrane transfer. Membranes were blocked with 5% milk followed by incubation with commercial Shh (Abcam 97029, 1:1000) and therapeutic test supernatant (1:20) antibodies. Blots were developed with ECL Reagents (Pierce).

### Cell lines and reagents

All cells were obtained from American Type Culture Collection (ATCC) and were cultured in ATCC-recommended media supplemented with 10% fetal bovine serum and 2% antibiotics (Penicillin-Streptomycin). Transient transfection of the pCMV-Shh plasmid (Origene) was introduced into 293T/A549 cells with Lipofectamine 2000 (Life Technologies) by following the manufacturer's recommendations.

### Flow cytometry screening and cell sorting

A549, 293T or 293T (+ pCMV Shh) cells were labeled with Shh antibody (Abcam 53281, 1:100) or therapeutic test antibodies (1:100) for 1 hour at room temperature after serum blocking. Following two PBS washes, a FITC-conjugated secondary antibody (Abcam 97029, 1:100) or an Alexa-Fluor 647 (Invitrogen, 1:1000) was used to label cells. Samples were screened by flow cytometry using an Accuri™ C6 machine (BD Biosciences). For fluorescence activated cell sorting, samples were sorted using an S3e™ Cell Sorter (Bio-Rad) and the Shh+ population was collected. For all experiments, cells processed without primary antibody served as a negative control. Experiments were performed in duplicates or triplicates.

### Immunoglobulin isotype determination

Immunoglobulin class (IgG, IgA, IgE, IgM) and subclass identities of screened and isolated antibodies were determined using cell culture supernatant with an isotyping kit by following the manufacturer's recommendations (Pierce™ Rapid Mouse Antibody Isotyping Kit plus Kappa and Lambda, Cat. No. 26179).

### Cell viability assay

Logarithmically growing cells were plated in antibiotic-free medium supplemented with 2% fetal bovine serum at a density of 5,000 cells per well in clear-bottom 96-well plates. The next day, cells were treated (triplicates) with increasing doses of in-house therapeutic antibodies (reconstituted in PBS) or PBS controls for 96 hours and subsequently assessed for cell viability by measuring ATP content with CellTiter-Glo Luminescent Cell Viability Assay (Promega). Signal intensity was measured on a Glomax^TM^ 96 Microplate Luminometer and percent cell survival was calculated based on the reading of PBS control cells set as 100%.

### Antitumor efficacy in NSCLC mouse model

Seven-week-old female nude mice (Jackson Laboratories) received injections of 10 × 10^6^ NSCLC A549 cells in 50% matrigel in the flank region. To assess establishment of tumors, mice were examined 10 days after inoculation and were randomly segregated into 2 treatment groups (IgG control and Ab 1C11-2G4, *n* = 5 per group). Beased on published literature, 8 mg/kg therapeutic or control antibodies were administered intratumorally 3 times a week for approximately 3 weeks. Tumor volumes were calculated using the formula (V = L × W^2^) with recorded caliper measurements. At the end point, tumors were harvested,weighed and imaged. All animal procedures were performed under IACUC-approved protocols and guidelines.

### Immunofluorescence staining

Control IgG and Ab 1C11-2G4-treated A549 tumors harvested from mice were sectioned (5 μM) and stained with a commercially available Shh antibody (Abcam 53281, 1:100) or Ki-67 (Cell Signalling, 1:800) after acetone fixation, 0.2% Triton-X 100 permeabilization, and serum-blocking steps. A FITC-conjugated secondary antibody (1:1000) and VECTASHIELD DAPI (Vector Laboratories) mounting medium were used to stain sections before imaging at 20X magnification using a Zeiss AxioImager 2 fluorescence microscope. Captured images were processed via ImageJ software and representative images are presented.

### Real-time PCR

Antibody-treated tumors were harvested to extract total RNA using Trizol (Invitrogen) and cDNA was synthesized using reverse transcriptase from iScript's cDNA Synthesis Kit (Bio-Rad). Real-time quantitative reverse transcript-polymerase chain reaction (qRT-PCR) was performed with TaqMan^®^ Universal PCR Master Mix (Life Technologies) using gene-specific PCR primers for human Shh-FAM (Life Technologies). Triplicate samples were run on an AB7900HT Fast Real Time PCR System thermocycler (Applied Biosystems) and GAPDH was used as a housekeeping gene for normalization. Ct values were analyzed using the 2^–∆ CT^ method and the data is presented as a fold change in target gene expression ± standard deviation.

### TUNEL assay

Promega's DeadEnd™ Fluorometric TUNEL System kit (G3250) was used to assess apoptosis in lung tumor sections according to the manusfacturer's instructions. The slides were then washed 3 times in PBS, and mounted in Vectashield^®^ with DAPI for analysis using a Zeiss Axio Imager 2 fluorescent microscope. Representative images for control IgG and Ab 1C11-2G4-treated tumors are presented.

### Apoptosis detection by annexin V and propidium iodide staining

A549 cells were treated with Ab 1C11-2G4 for 15 days or left untreated. Following two washes with ice-cold PBS, cells were stained using a FITC Annexin V/PI Apoptosis Detection kit (BD Pharmingen) following the manufacturer's recommendations. Samples were analyzed by flow cytometry and the resulting data was presented as a graph.

### Plasmids, transfections and luciferase assays

Transfections and luciferase assays were performed as previously described [42, 43].

### Statistical analyses

All experiments were repeated a minimum of 2 times with duplicate or triplicate samples. Values including controls are expressed as the mean ± SD. A 2-tailed paired Student's *t* test was used to assess differences between groups. Differences with a *p* value ≤ 0.05 were considered statistically significant.

## SUPPLEMENTARY MATERIALS FIGURES



## Abbreviations

Shh: Sonic Hegehog
mAb: monoclonal antibody
Ab: antibody
C: carboxyN: amino
term: terminus/terminal
ELISA: enzyme-linked immunosorbent assay
FACS: fluorescence-activated cell sorting
FDA: Food and Drug Administration
CSC: cancer stem cell
PK/PD: pharmacokinetic/pharmacodynamic
NSCLC: non-small cell lung cance
KLH: keyhole limpet hemocyanin
IV: intravenous
IP: intraperitoneal
IND: investigational new drug
BLI: biolayer inferometry
IgG: immunoglobulin G
Kd: dissociation constant
IF: immunofluoresence
FITC: fluorescein isothiocyanate
DAPI: 4′, 6-diamidino-2-phenylindole
qRT-PCR: quantitative real time polymerase chain reaction
cDNA: complementary deoxyribonucleic acid
GAPDH: glyceraldehyde 3-phosphate dehydrogenase
