# Supplementary material for: Preclinical characterization of therapeutic antibodies targeted at the carboxy-terminus of Sonic hedgehog
Source: Oncotarget. 2018 Feb 16;9(18):14311–23. doi: 10.18632/oncotarget.24510 (PMC5865672; doi:10.18632/oncotarget.24510)
Supplement: Supplementary file 1 [file oncotarget-09-14311-s001.pdf]

# Preclinical characterization of therapeutic antibodies targeted at the carboxy-terminus of Sonic hedgehog

## SUPPLEMENTARY MATERIALS

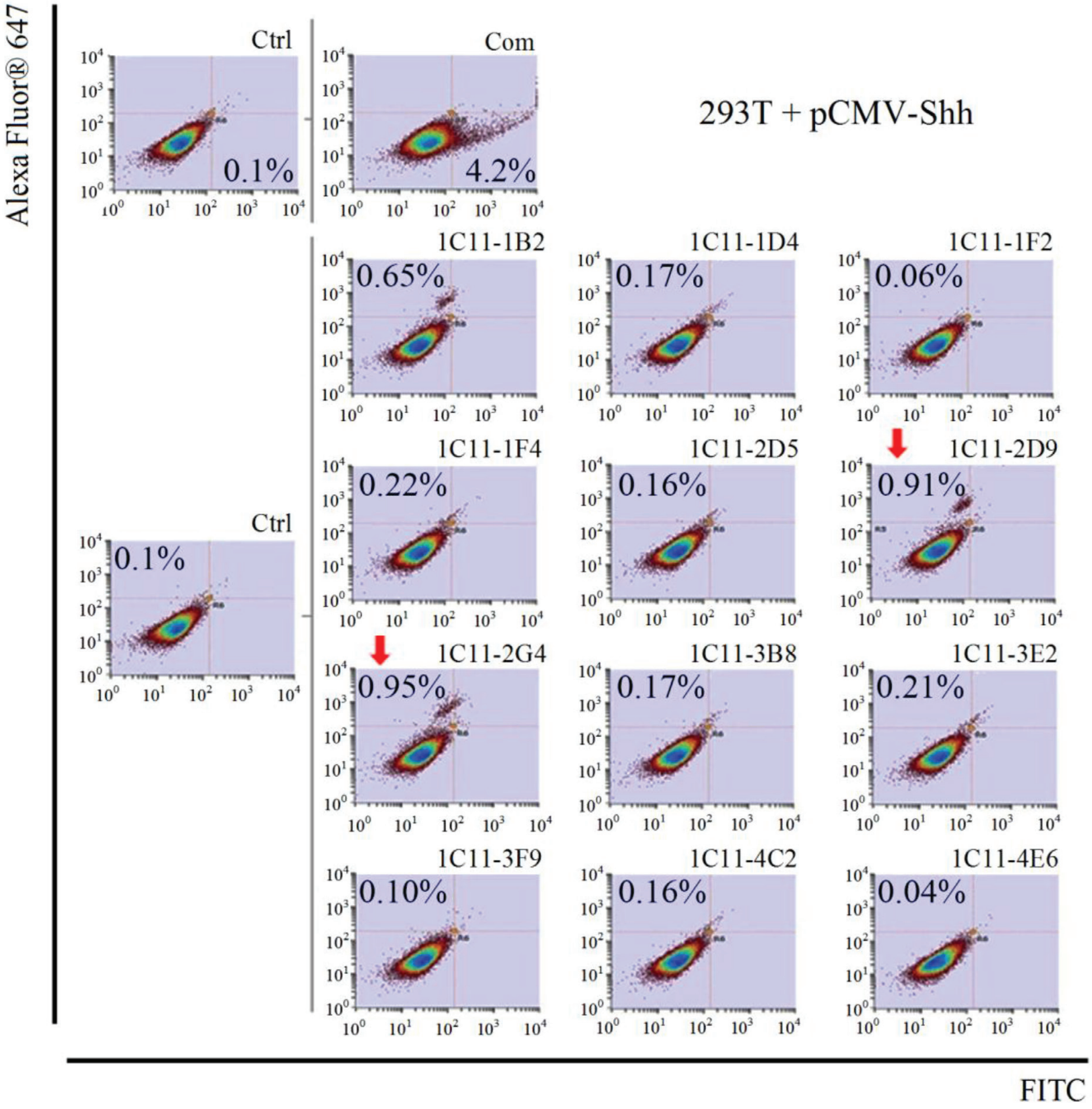


**Supplementary Figure 1: Screening of candidate therapeutic anti-Shh antibodies directed at the C-terminal of the Sonic Hedgehog protein.** Flow cytometric evaluation of Shh recognition performed with clonal supernatants incubated with 293T cells transfected with exogenous Shh. Twelve sub-clones of 1C11 were tested and 2D9 and 2G4 detected the highest percentage of cell-surface Shh compared with the other clones. The commercial (Com) antibody was used as a positive control. The secondary antibody alone incubated with cells was run as a negative control (Ctrl.).

A

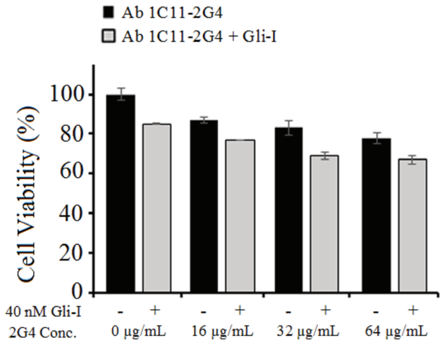


| Antibody Conc. | Ab 1C11-2G4 (%) | Ab 1C11-2G4 + Gli-I (%) |
|---|---|---|
| 0 µg/mL | 100% | 85% |
| 16 µg/mL | 87% | 77% |
| 32 µg/mL | 83% | 69% |
| 64 µg/mL | 78% | 67% |

Note: 40 nM Gli-I

B

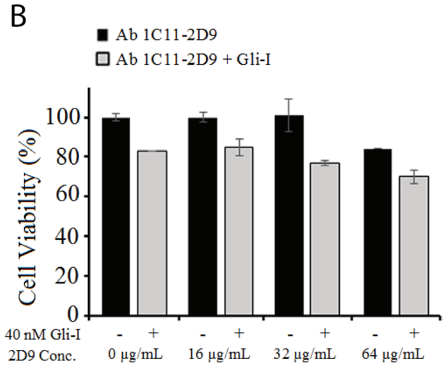


| Antibody Conc. | Ab 1C11-2D9 (%) | Ab 1C11-2D9 + Gli-I (%) |
|---|---|---|
| 0 µg/mL | 100% | 83% |
| 16 µg/mL | 100% | 85% |
| 32 µg/mL | 100% | 77% |
| 64 µg/mL | 84% | 70% |

Note: 40 nM Gli-I

C

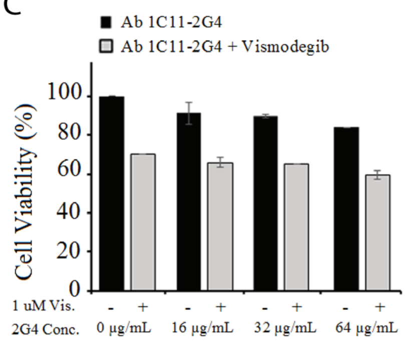


| Antibody Conc. | Ab 1C11-2G4 (%) | Ab 1C11-2G4 + Vismodegib (%) |
|---|---|---|
| 0 µg/mL | 100% | 70% |
| 16 µg/mL | 91% | 66% |
| 32 µg/mL | 90% | 65% |
| 64 µg/mL | 84% | 59% |

Note: 1 µM Vismodegib

D

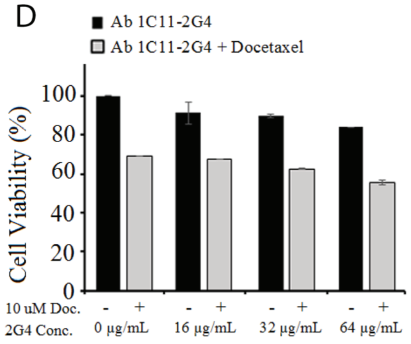


| Antibody Conc. | Ab 1C11-2G4 (%) | Ab 1C11-2G4 + Docetaxel (%) |
|---|---|---|
| 0 µg/mL | 100% | 69% |
| 16 µg/mL | 91% | 67% |
| 32 µg/mL | 90% | 63% |
| 64 µg/mL | 84% | 56% |

Note: 10 µM Docetaxel

**Supplementary Figure 2: Combinatorial regimens of C-terminal directed anti-Shh along with targeted therapies or chemotherapy result in increased efficacy.** NSCLC A549 cells were treated with indicated doses of C-term anti-Shh antibodies and combined with targeted therapies [Ab 1C11-2G4 + Gli-I (**A**) or Ab 1C11-2D9 + Gli-I (**B**) or Ab 1C11-2G4 + Vismodegib (**C**)] or chemotherapy [Ab 1C11-2G4 + Docetaxel (**D**)] in triplicate for 96 hours. The cell viability/ATP content was measured using CellTiter-Glo Luminescent Cell Viability Assay.

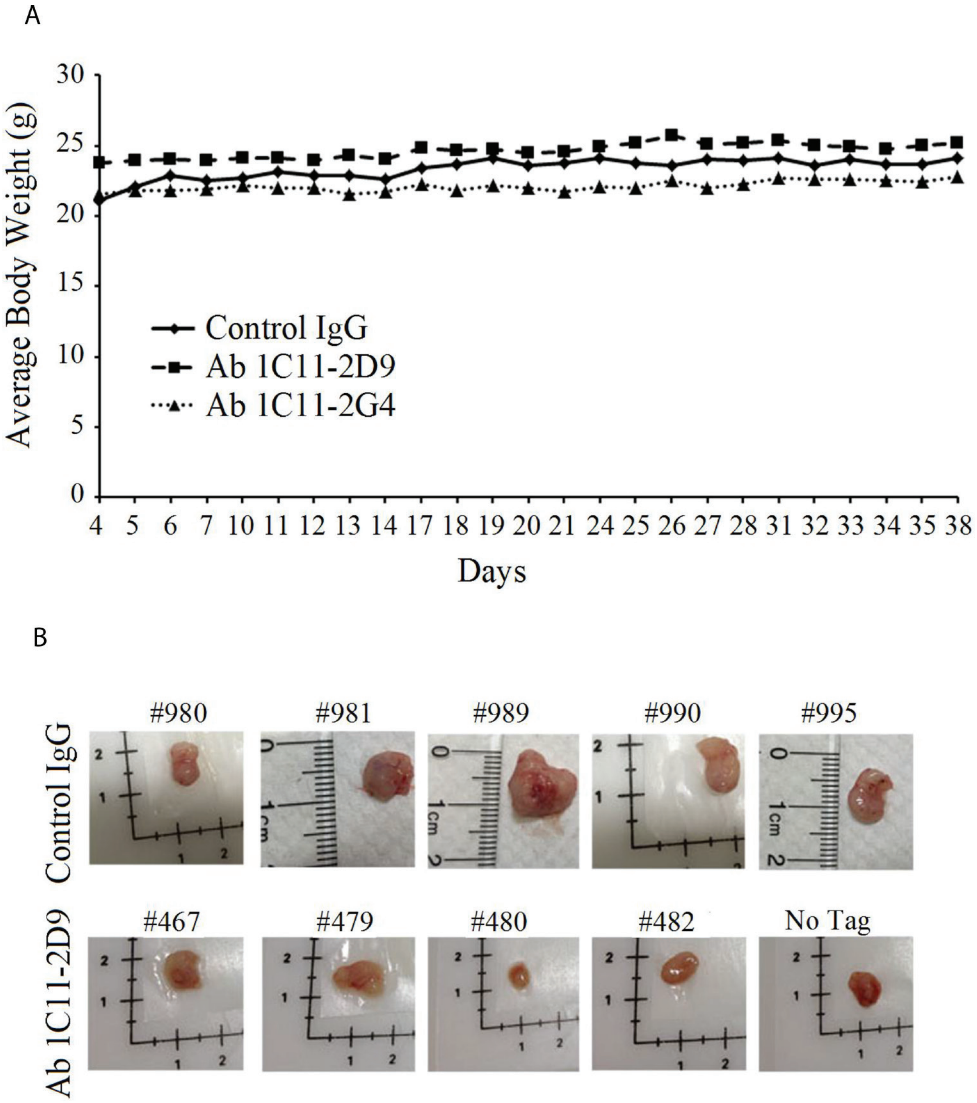


**Supplementary Figure 3: *in vivo* studies of C-term Shh Ab 1C11-2G4 and Ab 1C11-2D9 in lung NSCLC.** (**A**) Average body weight of all mice treated with Ab 1C11-2G4, Ab 1C11-2D9 or control IgG over the course of the study (38 days). (**B**) Images of tumors collected from control IgG antibody-treated or Ab 1C11-2D9 antibody-treated mice.
